# Tolerance to neurochemical and behavioral effects of the hallucinogen 25I-NBOMe

**DOI:** 10.1007/s00213-021-05860-5

**Published:** 2021-05-25

**Authors:** Monika Herian, Mateusz Skawski, Adam Wojtas, Małgorzata K. Sobocińska, Karolina Noworyta, Krystyna Gołembiowska

**Affiliations:** Department of Pharmacology, Polish Academy of Sciences, Maj Institute of Pharmacology, 12 Smętna, 31-343 Kraków, Poland

**Keywords:** 25I-NBOMe, Tolerance, Neurotransmitter release, Behavior

## Abstract

**Rationale:**

4-Iodo-2,5-dimethoxy-N-(2-methoxybenzyl)phenethylamine (25I-NBOMe) is a potent serotonin 5-HT_2A/2C_ receptor agonist with hallucinogenic activity. There is no data on the 25I-NBOMe effect on brain neurotransmission and animal performance after chronic administration.

**Objectives:**

We examined the effect of a 7-day treatment with 25I-NBOMe (0.3 mg/kg/day) on neurotransmitters’ release and rats’ behavior in comparison to acute dose.

**Methods:**

Changes in dopamine (DA), serotonin (5-HT), acetylcholine (ACh), and glutamate release were studied using microdialysis in freely moving rats. The hallucinogenic activity was measured in the wet dog shake (WDS) test. The animal locomotion was examined in the open field (OF) test, short-term memory in the novel object recognition (NOR) test. The anxiogenic/anxiolytic properties of the drug were tested using the light/dark box (LDB) test.

**Results:**

Repeated administration of 25I-NBOMe decreased the response to a challenge dose of DA, 5-HT, and glutamatergic neurons in the frontal cortex as well as weakened the hallucinogenic activity in comparison to acute dose. In contrast, striatal and accumbal DA and 5-HT release and accumbal but not striatal glutamate release in response to the challenge dose of 25I-NBOMe was increased in comparison to acute treatment. The ACh release was increased in all brain regions. Behavioral tests showed a motor activity reduction and memory deficiency in comparison to a single dose and induction of anxiety after the drug’s chronic and acute administration.

**Conclusions:**

Our findings suggest that multiple injections of 25I-NBOMe induce tolerance to hallucinogenic activity and produce alterations in neurotransmission. 25I-NBOMe effect on short-term memory, locomotor function, and anxiety seems to be the result of complex interactions between neurotransmitter pathways.

**Supplementary Information:**

The online version contains supplementary material available at 10.1007/s00213-021-05860-5.

## Introduction

Humans have used psychedelics (also known as serotonergic hallucinogens) for thousands of years. Nonetheless, scientists became interested in these substances only after Albert Hofmann discovered the psychoactive properties of lysergic acid diethylamide (LSD) in 1943 (Begola and Schillerstrom 2019). Although hallucinogens are derived from multiple structural families, they are known as powerful agents producing profound changes in consciousness, perception, and mood (Halberstadt 2015). According to their chemical backbone, psychedelics can be divided into the following categories: mescaline-like phenylalkylamines, psilocybin-like tryptamines, and a small subclass of LSD-like ergolines (Fantegrossi et al. 2008). Serotonin receptors mediate their action. Phenylalkylamines display a high binding affinity mainly for not only 5-HT_2A_ receptors but also 5-HT_2C_ and 5-HT_1A_ receptors, while tryptamines and ergolines are less selective for 5-HT receptor subtypes (Pierce and Peroutka 1989). 5-HT_2A_ receptors are primarily distributed in the cerebral cortex, especially on the apical dendrites of pyramidal cells in layer V (Weber and Andrade 2010). Several studies confirm that increased cortical release of glutamate is a common hallucinogen action mechanism (Herian et al. 2019; Muschamp et al. 2004). By enhancing glutamate release, 5-HT_2A_ receptors may control cognitive processes, such as attention, executive functions, and working memory (Mirjana et al. 2004). Through activation of the cortical 5-HT_2A_ receptors, psychedelics evoke head twitch behavior in rodents, which corresponds to hallucinations in humans (Glennon et al. 1984). In contrast, 5-HT_2C_ and 5-HT_1A_ receptors negatively contribute to the induction of head twitches (Klein et al. 2018).

Since 2010, a new class of powerful synthetic N-(2-methoxybenzyl)-2,5-dimethoxyphenethylamine (NBOMe) hallucinogens has been present on the drug market and has been used as a legal substitute for LSD (UNODC 2016). The iodine derivative, 4-iodo-2,5-dimethoxy-N-(2-methoxybenzyl)phenethylamine (25I-NBOMe), is one of the three NBOMe representatives most readily available to drug users (Lawn et al. 2014). Like other psychedelics, 25I-NBOMe acts as 5-HT_2A_ and 5-HT_2C_ receptor agonist due to its high in vitro binding affinity for these receptors (*K*_i_ = 0.6 and 4.6 nM, respectively) (Rickli et al. 2015). Many studies report an inverted U-shaped dose dependence of hallucinogenic activity in rodents measured with head and body twitch counts (Elmore et al. 2018; Herian et al. 2019). Apart from head and body twitch response, 25I-NBOMe also induced back muscle contractions, a 5-HT_2A_-mediated behavior in rats (Elmore et al. 2018). Moreover, it caused a time-dependent and dose-dependent inhibitory effect on locomotor activity in male Swiss-Webster (Gatch et al. 2017) and C57BL/6 J mice (Halberstadt 2017). Emerging evidence suggests that hallucinogens may have therapeutic efficacy in treating specific psychiatric disorders, such as post-traumatic stress disorder, alcohol and drug addiction (Bogenschutz and Ross 2018), anxiety disorders (Grob et al. 2011), and drug-resistant depression (Carhart-Harris et al. 2017). 25I-NBOMe seems to affect dopaminergic transmission in the rat nucleus accumbens shell and medial prefrontal cortex (Miliano et al. 2019). There is scarce data on the effect of chronic administration of NBOMe on neurotransmission and behavior in animal models. However, it has been shown that after repeated administration, other hallucinogens, such as LSD, DOM, and 25CN-NBOH, produce tolerance by downregulation of the 5-HT_2A_ receptor (Buchborn et al. 2018; Buckholtz et al. 1990; Gresch et al. 2005). Furthermore, it has also been demonstrated that rewarding and reinforcing effects of 25I-NBOMe, 25B-NBOMe, and 25N-NBOMe on mice are related to the dopaminergic system, as changes in expression of dopamine D_1_ and D_2_ receptors, dopamine transporter DAT, tyrosine hydroxylase, and dopamine (DA) levels were found in the nucleus accumbens and ventral tegmental area (VTA) (Custodio et al. 2019; Jeon et al. 2019; Seo et al. 2019).

Our earlier study showed that 25I-NBOMe administered in a wide range of doses affected extracellular neurotransmitter levels in the frontal cortex and induced head twitches (Herian et al. 2019). The latter effect seemed to be related to an increased release of glutamate in response to 5-HT_2A_ receptor stimulation located on pyramidal cells. However, the effect of a long-term administration of 25I-NBOMe on brain neurotransmission and animal behavior has not been researched yet.

Therefore, this study aimed at determining the effect of chronic administration of 25I-NBOMe on neurotransmission in several brain regions and on behavior in comparison to acute treatment. The release of DA, serotonin (5-HT), and glutamate in the rat frontal cortex, striatum, and nucleus accumbens was studied using microdialysis in freely moving animals. Since acetylcholine (ACh) is mainly engaged in memory and learning processes, its level was also assessed in certain rat brain regions. Locomotor activity was assessed in the open field test, cognitive functions in the novel object recognition test, and anxiogenic/anxiolytic effect was examined in the light/dark box test. In addition, drug-elicited wet dog shake response was also evaluated.

## Material and methods

### Animals

Adult male Wistar-Han rats (280–350 g; Charles River, Germany) were used in all experiments. The animals were initially acclimatized and housed (6 per cage) in environmentally controlled rooms (ambient temperature 23 ± 1 °C, humidity 55 ± 10%, and 12:12 light:dark cycle). Rats were handled once daily before the beginning of the experiments; an enriched environment was not applied. Animals had free access to tap water and typical laboratory food (VRF 1, Special Diets Services, UK).

### Drugs and reagents

4-Iodo-2,5-dimethoxy-N-(2-methoxybenzyl)phenethylamine hydrochloride (25I-NBOMe) was purchased from Chiron AS (Norway); ketamine and xylazine hydrochlorides from Biowet Puławy (Poland). All necessary chemicals of the highest purity used for analysis by high-performance liquid chromatography (HPLC) were obtained from Merck (Poland). O-phthalaldehyde (OPA) of Sigma-Aldrich (Poland) was used for the derivatization of glutamate to an electroactive compound.

### Drug administration

The subcutaneous (sc) injection of 25I-NBOMe was chosen because it seems to be a suitable route of administration in the case of this compound compared to intraperitoneal injection, as shown by Halberstadt (2017). The dosage was chosen estimated based on our previous experiments (Herian et al. 2019), as the dose of 0.3 mg/kg was eliciting a more subtle yet profound effect in comparison to higher doses, which seemed favorable for chronic administration. Moreover, the NBOMes are connected with increased toxicity in humans (Halberstadt 2017); therefore, the dose of 0.3 mg/kg was chosen to prevent the possible toxic effects in rats. To ensure the development of possible tolerance, the 7-day dosing regimen of consecutive injections was selected.

**Scheme 1:**
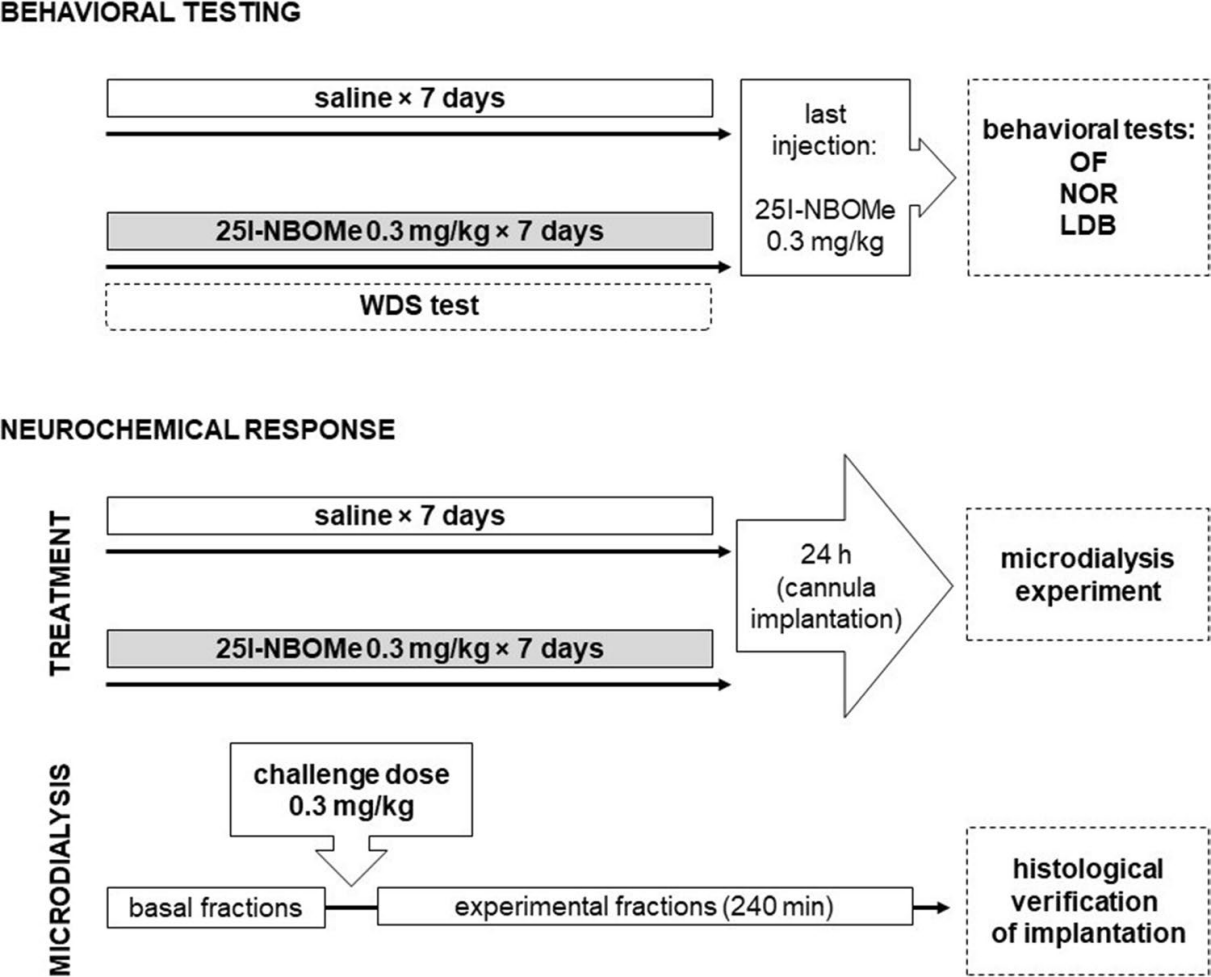
Schematic presentation of drug treatments

One group of animals received once-daily sc injections of 25I-NBOMe at a dose of 0.3 mg/kg/day for 7 days. 25I-NBOMe was dissolved in 0.9% NaCl. The second group received 0.9% NaCl solution in the same manner. The next day, both experimental groups were administered a 0.3 mg/kg dose of 25I-NBOMe. The behavioral tests were conducted 20 min after the injection. Another cohort of rats was treated with 25I-NBOMe at a dose of 0.3 mg/kg/day or 0.9% NaCl solution for 7 days. Animals were implanted with microdialysis probes. The next day, microdialysis experiments were performed. Schematic presentation of drug treatments is presented in Scheme 1.

### Brain microdialysis

A total of 75 mg/kg and 10 mg/kg of ketamine and xylazine, respectively, were injected intramuscularly to anesthetize animals. Microdialysis probes (MAB 4.15.3Cu, MAB 4.15.4Cu, AgnTho’s AB, Sweden) were implanted into the following brain structures using the determined coordinates (mm): frontal cortex AP + 2.7, L + 0.8, V − 6.5, striatum AP + 1.2, L + 2.8, V − 7.0, and nucleus accumbens AP + 1.6, L + 1.0, V − 8.0; from the dura (Paxinos and Watson 1998). The implantation of microdialysis probes in rats receiving multiple injections was performed after the last injection. The following day, probe inlets were connected to a syringe pump (BAS, West Lafayette, IN, USA) which delivered artificial cerebrospinal fluid composed of the following (mM): 147 NaCl, 4 KCl, 2.2 CaCl_2_·2H_2_O, and 1.0 MgCl_2_ at a flow rate of 2 µL/min. Five baseline samples were collected every 20 min after the washout period of 2 h. A challenge dose (0.3 mg/kg) of 25I-NBOMe was administered, and dialysate fractions were collected for the next 240 min. As the experiment ended, the rats were terminated and their brains underwent histological examination to validate the probe placement.

### Extracellular concentration of DA, 5-HT, ACh, and glutamate

Extracellular DA and 5-HT levels were analyzed by HPLC with electrochemical detection. Chromatography was performed using an Ultimate 3000 System (Dionex, USA), electrochemical detector Coulochem III (model 5300; ESA, USA) with a 5020 guard cell, a 5040 amperometric cell, and a Hypersil Gold C18 analytical column (3 μm, 100 × 3 mm; Thermo Fisher Scientific, USA). The mobile phase consisted of 0.1 M KH_2_PO_4_ buffer at pH 3.8, 0.5 mM Na_2_EDTA, 100 mg/L 1-octanesulfonic acid sodium salt, and 2% methanol. The flow rate during analysis was set at 0.6 mL/min, and the applied potential of a guard cell was 600 mV, whereas the amperometric cell was 300 mV with a sensitivity set at 10 nA/V. The chromatographic data were processed by Chromeleon v.6.80 (Dionex) software package. The detection limit in dialysates was 0.002 pg/10 μL for DA and 0.01 pg/10 μL for 5-HT.

Extracellular levels of ACh were analyzed by UHPLC with electrochemical detection. The ACh analysis is based on ion-pairing HPLC separation, followed by on-line enzymatic conversion of ACh to hydrogen peroxide and detection on a Pt working electrode (SenCell with 2 mm Pt working electrode) and HyREF reference electrode at the potential of 200 mV. Chromatography was performed using the ALEXYS Neurotransmitter Analyzer, a DECADE Elite electrochemical detector, AS 110 Autosampler, and LC 110 pump (Antec Leyden B. V., Zoeterwoude, The Netherlands). ACh as positively charged was separated on Acquity UPLC HSS T3 analytical column (1.8 μm, 1 × 50 mm; Waters, Milford, MA, USA). After separation, ACh passed through an immobilized enzyme reactor AChE/ChOx IMER (AC-ENZYM II, 1 × 4 mm, Eicom, Kyoto, Japan). The mobile phase was composed of 50 mM monosodium orthophosphate buffer adjusted to pH 7.8, 0.5 mM Na_2_EDTA, 2.8 g/L 1-octanesulfonic acid sodium salt, and 0.5 mM tetramethylammonium chloride. The flow rate during analysis was set to 0.05 mL/min. The chromatographic data were processed by the CLARITY v.6.2.0.208 (DataApex Ltd.) chromatography software run on a personal computer. The detection limit of ACh in dialysates was 1.1 nM.

Glutamate levels in the extracellular fluid were measured electrochemically after derivatization with OPA/sulfite reagent to form isoindole-sulfonate derivative (Rowley et al. 1995). Chromatography was performed using an Ultimate 3000 pump (Dionex), LC-4B amperometric detector with a cross-flow detector cell (BAS), and an HR-80 column (3 μm, 80 × 4.6 mm; ESA, USA). The mobile phase was composed of 100 mM monosodium orthophosphate at pH 4.6 and 4% methanol. The flow rate during analysis was set to 1 mL/min and the applied potential of a 3-mm glassy carbon electrode was set at + 600 mV at a sensitivity of 5 nA/V. The glutamate-derivative peak was compared with the respective standard, and the data were processed using the Chromax 2005 (Pol-Lab, Poland) software. The detection limit of glutamate in dialysates was 0.03 ng/10 μL.

### Wet dog shake test

The rats’ behavior, defined as rapid shaking of the head, neck, and trunk from one side to the other, resembling a wet dog shaking to dry itself, is called as wet dog shake (WDS). The WDS test was carried out based on the procedure reported by Nagayama and Lu (1996). Measurements of WDS were conducted for 80 min after each injection and were expressed as an average of sum values of all episodes during the observation time.

### Open field test

The open field (OF) test was based on the procedure described by Rogoz and Skuza (2011). The laboratory room was dark, and only the center of the open field was illuminated with a bright light (150 lx) above the platform. Rats were placed in the middle of the black round arena (1 m in diameter, divided into 8 sections). During a 10-min test, rats’ exploratory activity, expressed as the time of walking, a number of sector line crossings, and the episodes of peeping under the edge of the arena, was measured.

### Novel object recognition test

The procedure of the novel object recognition (NOR) test was performed according to Antunes and Biala (2012) and Orzelska-Gorka et al. (2016). An apparatus consisted of a wooden box with black-painted walls (60 × 60 × 40 cm) and a bright light (150 lx) focused on the center. Animals were left to acclimate to the new environment 24 h before the testing. On the day of the experiment, rats were habituated to a dimly lit experimental room for at least 1 h before the procedure. The NOR test consisted of an introductory and recognition session (5 min each) with a 30-min inter-session interval. The introductory session was performed with two identical objects (A1 and A2) situated in opposite corners. During the recognition session, one object was replaced with a novel one (A = familiar, B = novel). The location of a novel object in the recognition session was randomly assigned to each rat. The novel objects were used interchangeably in each experimental group. The object exploration was defined as sniffing or touching with one’s nose and/or forepaws. The exploration time was measured using a digital laboratory timer. The arena and the objects were cleaned between each session. Recognition index (*Ri*) was calculated as the time of exploration of a novel object relative to the total exploration time of both objects. The “*Ri*” ratio over 50% was defined as successful discrimination.

### Light/dark box test

The light/dark box (LDB) test was performed using four computer-controlled Seamless Open Field Arenas for rats (43 × 43 × 30 cm; Med Associates, USA) with 16 infrared emitters and photodetectors on each side of the box. The procedure of Noworyta-Sokolowska et al. (2019) was adapted to the experimental design. A dark insert with a hole divided the chamber into two equally sized compartments: a light compartment and a dark one. An additional light source (220 lm) was placed above the light compartment to make it more anxiogenic. Animals were placed in the dark compartment and were allowed to explore the arena freely for 15 min. The measured parameters included ambulatory distance, vertical and stereotypic activity time, and the time spent in the dark and light compartment. The data were collected using the Med State software (activity monitor, Med Associates).

### Data analysis

Drug effects on DA, 5-HT, ACh, and glutamate release in the brain regions were analyzed with repeated measures ANOVA or one-way ANOVA, where appropriate, on normalized responses followed by Tukey’s post hoc test. All obtained data were presented as a percent of the basal level assumed to be 100%. The WDS test, locomotor behavior of rats in the OF test, and cognitive functions in the NOR test were analyzed using one-way ANOVA followed by Tukey’s post hoc test. The data collected from the LDB test were analyzed using Mann–Whitney *U* test or Wilcoxon *T* test. The differences were considered significant if *p* < 0.05. The detected outliers were removed from the data set using Grubb’s test. All statistical analyses were carried out using STATISTICA v.10 StatSoft Inc. 1984–2011 (USA) and GraphPad Prism v.5.00 GraphPad Software Inc. (USA).

## Results

### The effect of acute and chronic administration of 25I-NBOMe on the extracellular levels of DA, 5-HT, ACh, and glutamate in the rat frontal cortex

**Fig. 1:**
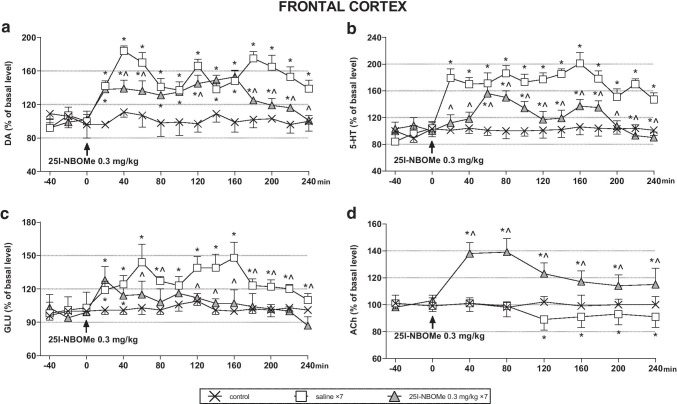
The time-course effect of 25I-NBOMe on extracellular levels of dopamine (DA), serotonin (5-HT), glutamate (GLU), and acetylcholine (ACh) in the rat frontal cortex (**a**-**d**), respectively. Values are the mean ± standard error of the mean (SEM), *n* = 6 per experimental group. The drug injection is indicated with an arrow. * *p* < 0.01 vs. control group; ^ *p* < 0.01 saline vs. repeated 25I-NBOMe administration (repeated measures ANOVA and Tukey’s post hoc test)

25I-NBOMe significantly increased extracellular levels of DA, 5-HT, and glutamate in the rat frontal cortex (Fig. 1a, b, c). However, the response of DA, 5-HT, and glutamate systems to a challenge dose of 0.3 mg/kg in animals treated repeatedly for 7 days with 25I-NBOMe was weaker in comparison to acutely injected rats. Repeated measures ANOVA showed an effect of treatment (*F*_2,15_ = 457, *p* < 0.0001) and time (*F*_11,165_ = 7.1, *p* < 0.0001) on DA levels, and a time × treatment interaction (*F*_22,165_ = 8.3, *p* < 0.0001). There was an effect of treatment (*F*_2,15_ = 600, *p* < 0.0001) and time (*F*_11,165_ = 29, *p* < 0.0001) on 5-HT levels, and a time × treatment interaction (*F*_22,165_ = 14, *p* < 0.0001). Similarly, there was an effect of treatment (*F*_2,15_ = 119, *p* < 0.0001) and time (*F*_11,165_ = 13, *p* < 0.0001) on glutamate levels, and a time × treatment interaction (*F*_22,165_ = 7.9, *p* < 0.0001). In contrast, extracellular ACh levels increased in response to a challenge dose of 0.3 mg/kg in animals treated repeatedly for 7 days with 25I-NBOMe in comparison to control and acutely treated group (Fig. 1d). Repeated measures ANOVA showed an effect of treatment (*F*_2,15_ = 1254, *p* < 0.0001) and time (*F*_5,75_ = 16, *p* < 0.0001) on ACh levels, and a time × treatment interaction (*F*_10,75_ = 6.8, *p* < 0.0001).

### The effect of acute and chronic administration of 25I-NBOMe on the extracellular levels of DA, 5-HT, ACh, and glutamate in the rat striatum

**Fig. 2:**
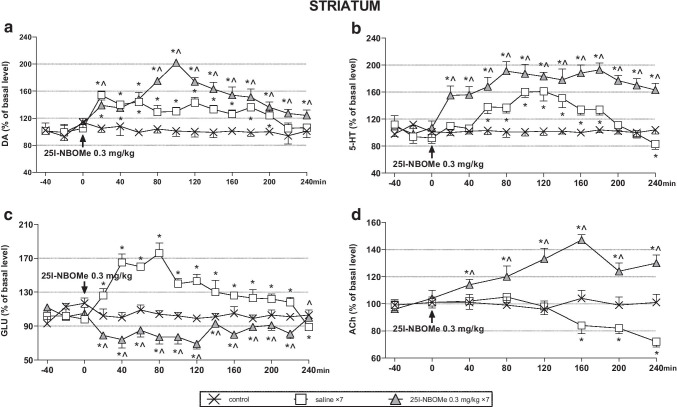
The time-course effect of 25I-NBOMe on extracellular levels of dopamine (DA), serotonin (5-HT), glutamate (GLU), and acetylcholine (ACh) in the rat striatum (**a**-**d**), respectively. Values are the mean ± standard error of the mean (SEM), *n* = 6 per experimental group. The drug injection is indicated with an arrow. * *p* < 0.01 vs. control group; ^ *p* < 0.01 saline vs. repeated 25I-NBOMe administration (repeated measures ANOVA and Tukey’s post hoc test)

The enhancement in DA and 5-HT extracellular levels in response to a challenge dose of 0.3 mg/kg was stronger in animals treated repeatedly with 25I-NBOMe than in acutely injected rats (Fig. 2a, b). In contrast, the glutamatergic response to the challenge dose decreased in animals receiving repeated doses of 25I-NBOMe in comparison to acutely injected rats (Fig. 2c). Repeated measures ANOVA showed an effect of treatment (*F*_2,15_ = 251, *p* < 0.0001) and time (*F*_11,165_ = 33, *p* < 0.0001) on DA levels, and a time × treatment interaction (*F*_22,165_ = 20, *p* < 0.0001). There was an effect of treatment (*F*_2,15_ = 1136, *p* < 0.0001) and time (*F*_11,165_ = 25, *p* < 0.0001) on 5-HT levels, and a time × treatment interaction (*F*_22,165_ = 12, *p* < 0.0001). Similarly, there was an effect of treatment (*F*_2,15_ = 256, *p* < 0.0001), and time (*F*_11,165_ = 19, *p* < 0.0001) on glutamate levels, and a time × treatment interaction (*F*_22,165_ = 37, *p* < 0.0001). The response of ACh neurons to a challenge dose of 0.3 mg/kg increased in animals treated repeatedly for 7 days with 25I-NBOMe in comparison to control and acutely injected group (Fig. 2d). Repeated measures ANOVA showed an effect of treatment (*F*_2,15_ = 447, *p* < 0.0001) and time (*F*_5,75_ = 10, *p* < 0.0001) on ACh levels, and a time × treatment interaction (*F*_10,75_ = 25, *p* < 0.0001).

### The effect of acute and chronic administration of 25I-NBOMe on the extracellular levels of DA, 5-HT, ACh, and glutamate in the rat nucleus accumbens

**Fig. 3:**
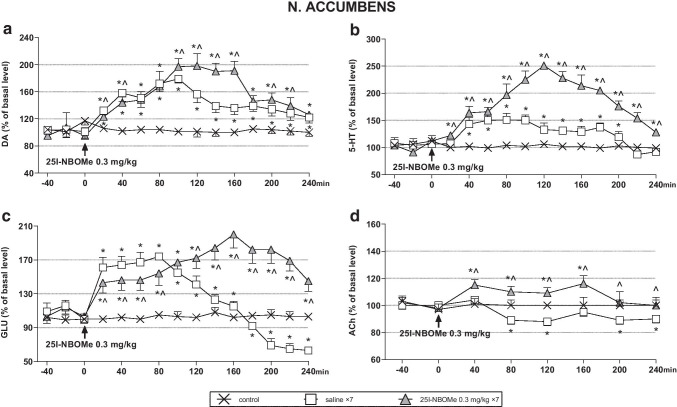
The time-course effect of 25I-NBOMe on extracellular levels of dopamine (DA), serotonin (5-HT), glutamate (GLU), and acetylcholine (ACh) in the rat nucleus accumbens (**a**-**d**), respectively. Values are the mean ± standard error of the mean (SEM), *n* = 6 per experimental group. The drug injection is indicated with an arrow. * *p* < 0.01 vs. control group; ^ *p* < 0.01 saline vs. repeated 25I-NBOMe administration (repeated measures ANOVA and Tukey’s post hoc test)

The response of DA, 5-HT, and glutamate neurons to a challenge dose of 0.3 mg/kg in animals treated repeatedly with 25I-NBOMe was more potent than in acutely injected rats (Fig. 3a, b, c). Repeated measures ANOVA showed an effect of treatment (*F*_2,15_ = 1173, *p* < 0.0001) and time (*F*_11,165_ = 86, *p* < 0.0001) on DA levels, and a time × treatment interaction (*F*_22,165_ = 47, *p* < 0.0001). There was also an effect of treatment (*F*_2,15_ = 952, *p* < 0.0001) and time (*F*_11,165_ = 57, *p* < 0.0001) on 5-HT levels, and a time × treatment interaction (*F*_22,165_ = 27, *p* < 0.0001). The treatment effect on glutamate levels was significant (*F*_2,15_ = 705, *p* < 0.0001), and there was also an effect of time (*F*_11,165_ = 31, *p* < 0.0001) and time × treatment interaction (*F*_22,165_ = 52, *p* < 0.0001). The response of ACh neurons to a challenge dose of 0.3 mg/kg increased in animals treated repeatedly for 7 days with 25I-NBOMe in comparison to acutely injected group (Fig. 3d). Repeated measures ANOVA showed an effect of treatment (*F*_2,15_ = 167, *p* < 0.0001) and time (*F*_5,75_ = 16, *p* < 0.0001) on ACh levels, and a time × treatment interaction (*F*_10,75_ = 4.8, *p* < 0.003).

### The total effect of acute and chronic administration of 25I-NBOMe on DA, 5-HT, ACh, and glutamate in the rat frontal cortex, striatum, and nucleus accumbens

**Fig. 4:**
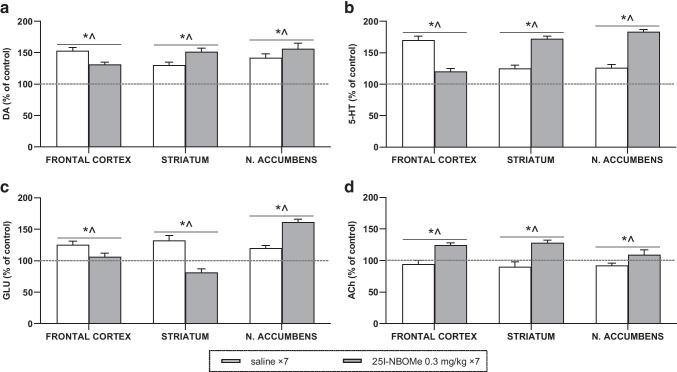
The total effect of 25I-NBOMe on extracellular levels of dopamine (DA) in **a**, serotonin (5-HT) in **b**, glutamate (GLU) in **c** and acetylcholine (ACh) in **d** in the rat frontal cortex, striatum, and nucleus accumbens calculated as an area under the concentration–time curve (AUC) and expressed as the percent of control. Values are the mean ± standard error of the mean (SEM), *n* = 6 per experimental group. * *p* < 0.01 vs. control group, ^ *p* < 0.01 saline vs. repeated 25I-NBOMe administration (one-way ANOVA and Tukey’s post hoc test)

The summary of the total effect of 25I-NBOMe on DA, 5-HT, ACh, and glutamate in the rat frontal cortex, striatum, and nucleus accumbens, calculated as an area under the curve (AUC) and expressed as a percent of each basal level, is presented in Fig. 4a, b, c, and d. A repeated administration of 25I-NBOMe caused a weaker increase in DA, 5-HT, and glutamate levels in the frontal cortex and glutamate in the striatum in response to the challenge dose of the drug in comparison to a single dose, whereas a more significant increase was observed in DA, 5-HT levels in the striatum and nucleus accumbens, glutamate in the nucleus accumbens, and ACh in all brain regions. Statistical data of one-way ANOVA analysis are presented in Table 1S (supplementary material).

### The effect of acute and chronic administration of 25I-NBOMe on the rats’ behavior

**Fig. 5:**
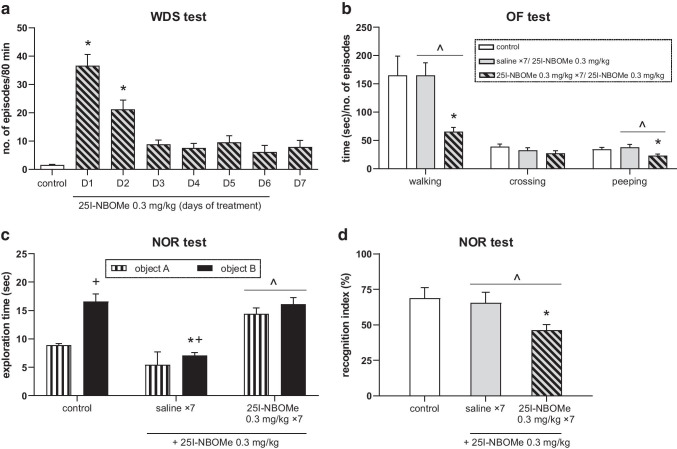
The effect of 25I-NBOMe on wet dog shake response (WDS), on locomotor behavior in the open field (OF) test, and on performance of rats in the novel object recognition (NOR) test. **a** The number of WDS episodes counted for 80 min starting immediately after the injection. Values are the mean ± standard error of the mean (SEM), *n* = 15 per experimental group. * *p* < 0.001 vs. control group (repeated measures ANOVA and Tukey’s post hoc test). **b** The time spent on walking, number of crossing episodes, and number of peeping episodes in the OF test. Values are the mean ± standard error of the mean (SEM), *n* = 11–12 per experimental group. * *p* < 0.01 vs. control group, ^ *p* < 0.05 vs. 25I-NBOMe 0.3 mg/kg (one-way ANOVA and Tukey’s post hoc test). **c** Exploration time in the recognition session for the familiar (A) and novel object (B) in the NOR test. **d** *Ri* expressed as a percent of the time spent on novel object exploration in relation to the total exploration time of both the novel and familiar objects. Values are the mean ± standard error of the mean (SEM), *n* = 9–11 per experimental group. * *p* < 0.01 vs. control; ^ *p* < 0.05 vs. 25I-NBOMe 0.3 mg/kg (one-way ANOVA and Tukey’s post hoc test); + *p* < 0.05 novel vs. familiar object (test *t*)

25I-NBOMe induced wet dog shake (WDS) response. The effect of repeated administration of 0.3 mg/kg 25I-BOMe dose for 7 days was declining in time and remained at a similar low level from day 3 until day 7 (Fig. 5a). Repeated measures ANOVA showed a significant effect of treatment (*F*_1,28_ = 47.3, *p* < 0.0001) and time (*F*_7,196_ = 34.8, *p* < 0.0001), and a time × treatment interaction (*F*_7,196_ = 34.3, *p* < 0.0001).

Locomotor activity of rats observed in the OF and expressed as the time of walking and the number of peeping episodes significantly decreased in groups treated repeatedly for 7 days with 0.3 mg/kg 25I-NBOMe dose in comparison to control and single-dose group (Fig. 5b). One-way ANOVA showed a significant effect of treatment for walking (*F*_2,31_ = 6.2, *p* < 0.005) and peeping (*F*_2,31_ = 9.2, *p* < 0.001), but not for crossing (*F*_2,31_ = 1.5, *p* > 0.05).

The time of novel object exploration compared with familiar object exploration during the NOR test recognition session was higher both in control and single dose-treated rats (Fig. 5c). However, the novel object exploration time in rats treated with a single dose of 25I-NBOMe was significantly shorter than in the control group. In contrast, animals treated repeatedly for 7 days with 25I-NBOMe (0.3 mg/kg) explored both familiar and new objects for a similar amount of time. One-way ANOVA showed a significant effect of treatment on exploring familiar object (*F*_2,28_ = 3.2, *p* < 0.05) and new object (*F*_2,28_ = 29, *p* < 0.001). There was a significant difference in the *Ri* between animals treated with the single dose of 0.3 mg/kg and repeated doses of 25I-NBOMe (*F*_2,28_ = 7.1, *p* < 0.003). Importantly, the *Ri* in the 25I-NBOMe-repeatedly treated group reached ca. 46.3% (Fig. 5d).

**Fig. 6:**
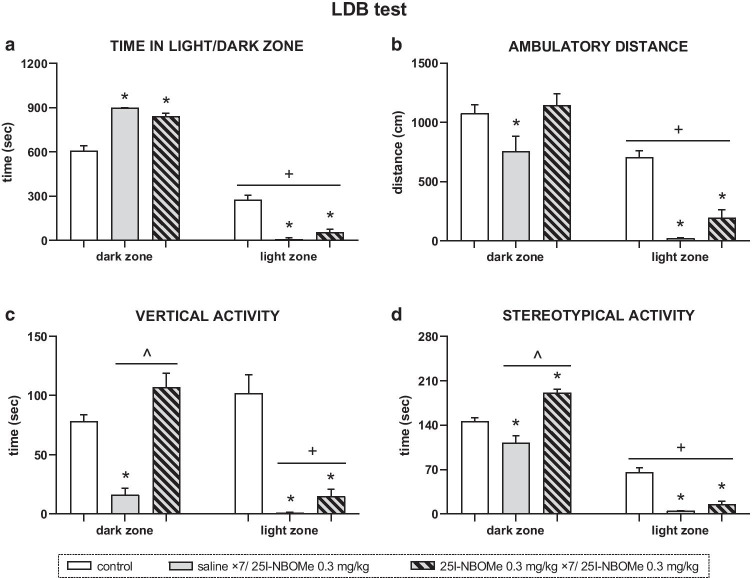
The effect of 25I-NBOMe on activity of rats in the light/dark box (LDB) test. **a** The time spent in the dark and light zone. **b**-**d** Ambulatory distance, vertical, and stereotypic activity, respectively in the dark and light zone. Values are the mean ± standard error of the mean (SEM), *n* = 11–13 per experimental group. * *p* < 0.04 vs. control, ^ *p* < 0.02 vs. 25I-NBOMe 0.3 mg/kg (Mann–Whitney *U* test), + *p* < 0.04 light vs. dark zone (Wilcoxon *T* test)

In the LDB test, the time spent in the dark compartment was longer than in the light zone for control animals (*T* =  − 108, *p* < 0.0008) and for rats administered a single dose (*T* =  − 21, *p* < 0.04) and chronic injections of 25I-NBOMe dose of 0.3 mg/kg (*T* =  − 28, *p* < 0.02) (Fig. 6a). The time spent in the dark zone in groups treated with the single and repeated doses of 0.3 mg/kg of 25I-NBOMe was similar, but longer than in control (*U* = 0, *U* = 2, respectively; *p* < 0.0001). On the contrary, the time spent in the light zone was significantly decreased in groups of rats given single dose (*U* = 0, *p* < 0.0001) or repeated doses of 0.3 mg/kg (*U* = 3, *p* < 0.0001) in comparison to control (Fig. 6a). Exploration of the dark zone, expressed as ambulatory distance, vertical, and stereotypical activity, significantly decreased by single 25I-NBOMe dose of 0.3 mg/kg (*U* = 18, *p* < 0.04; *U* = 1, *p* < 0.0001; *U* = 12, *p* < 0.009, respectively) in comparison to control, while in the groups treated repeatedly with 0.3 mg/kg of 25I-NBOMe, only the stereotypical activity time significantly increased with control rats (*U* = 2, *p* < 0.0001) (Fig. 6b, c, d). Moreover, ambulatory distance, as well as vertical and stereotypical activity time in the light zone, decreased after single dose (*U* = 0, *p* < 0.0001) and repeated doses of 0.3 mg/kg 25I-NBOMe (ambulatory distance: *U* = 3; vertical activity: *U* = 1; stereotypical activity: *U* = 2.5; *p* < 0.0001) in comparison to control rats. However, only in the dark zone, multiple injections of 25I-NBOMe significantly increased vertical activity time (*U* = 0, *p* < 0.02) and stereotypical activity time (*U* = 0, *p* < 0.0006) when compared with a single dose of 0.3 mg/kg. The ambulatory distance in the light compartment was shorter for control (*T* =  − 114, *p* < 0.0004), and for rats administered a single dose (*T* =  − 21, *p* < 0.04) and chronic injections of 25I-NBOMe (*T* =  − 28, *p* < 0.02) in comparison to the dark compartment. Similarly, stereotypical activity time in the light zone decreased in comparison to the dark zone in all groups of animals (control: *T* =  − 120, *p* < 0.0001; single dose: *T* =  − 21, *p* < 0.04; repeated doses: *T* =  − 28, *p* < 0.02). Furthermore, in the light compartment, vertical activity time significantly decreased for animals treated with single dose (*T* =  − 21, *p* < 0.04) and repeated doses of 25I-NBOMe (*T* =  − 28, *p* < 0.02), when comparing to the vertical activity in the dark compartment (Fig. 6b, c, d).

## Discussion

In the present work, we examined the effect of repeated administration of a low 25I-NBOMe dose on several neurotransmitters in the frontal cortex, striatum and nucleus accumbens, and hallucinogenic activity, locomotor activity, short-term memory, and anxiety in rats.

### Regulation of cortical glutamate, DA, and 5-HT release

In contrast to acute administration, we found that a 7-day exposure to 0.3 mg/kg of 25I-NBOMe led to the reduced response of DA, 5-HT, and glutamate neurons to a challenge dose of the drug in the frontal cortex and to the reduced WDS counts. These neurochemical and behavioral data indicate tolerance development observed from the third day of administration in the WDS test. The observed changes in the activity of neurotransmitters and animal behavior suggest the development of tolerance, which seems to result from 5-HT_2A_ receptors’ downregulation and their desensitization (Nichols 2016). Buckholtz et al. (1990) reported a decrease in 5-HT_2_ receptor density in the rat brain after multiple injections of LSD. Damjanoska et al. (2004) found that a 7-day treatment with DOI decreased 5-HT-stimulated PLC activity in the frontal cortex; it is suggested that desensitization of 5-HT_2A_ receptors is most likely attributable to post-translational modifications of the receptor due to a change in 5-HT_2A_ receptor coupling to G proteins.

This phenomenon is common to serotonergic hallucinogens, and close congeners in this class shared it: 25CN-NBOH or LSD and DOM (Buchborn et al. 2018; Buckholtz et al. 1990; Gresch et al. 2005). Multiple studies have demonstrated that cortical 5-HT_2A_ receptors are localized primarily in apical dendrites of pyramidal neurons with a minor localization to GABAergic interneurons (Miner et al. 2003; Willins et al. 1997). It is proposed that changes in glutamate release parallel hallucinogenic activity and this is a common mechanism in the action of hallucinogens (Herian et al. 2018; Herian et al. 2019; Muschamp et al. 2004; Noworyta-Sokolowska et al. 2016; Scruggs et al. 2003). This effect results from the activation of 5-HT_2A_ receptors located on cortical pyramidal cells since their blockade with the selective antagonist M100907 reduced WDS and cortical glutamate release (Herian et al. 2020; Scruggs et al. 2003). A rapid loss of 5-HT_2A_ receptor responsiveness seems to cause a desensitization of 5-HT_2A_ receptor-mediated glutamate release in the frontal cortex. Consequently, glutamatergic efferents which innervate dopaminergic and serotonergic cells projecting to the cortex (Alex and Pehek 2007; Di Matteo et al. 2008; Martin-Ruiz et al. 2001) may be responsible for the decreased release from cortical DA and 5-HT terminals observed in our study after repeated 25I-NBOMe treatment.

### Regulation of glutamate, DA, and 5-HT release in the striatum and nucleus accumbens

Immunohistochemical studies have indicated a low density of 5-HT_2A_ receptors in the VTA and substantia nigra (SN) presumably distributed on GABAergic cells (Alex and Pehek 2007). Furthermore, receptor binding studies demonstrated a high level of 5-HT_2A_ receptors in the striatum localized on afferents arising mainly from the cortex and globus pallidus (Bubser et al. 2001). Both striato-pallidal and striato-nigral neurons also express 5-HT_2A_ receptors (Ward and Dorsa 1996). In our study, DA and 5-HT neurons in the striatum and nucleus accumbens responded with increased strength to a challenge dose in animals pretreated repeatedly with 25I-NBOMe, in comparison to the acutely injected group. These data allow us to hypothesize that tolerant 5-HT_2A_ receptors located on efferent GABAergic neurons or VTA/SN GABAergic interneurons exert a weaker stimulating effect on GABA release. Thus, disinhibition of dopamine neurons from GABAergic control may result in higher extracellular levels of DA in response to a challenge dose observed in the striatum and nucleus accumbens. These findings are in line with a recent study by Custodio et al. (2019), who demonstrated that 7 days of treatment with a close congener of this class of compound, 25B-NBOMe, increased DA level in the nucleus accumbens of mice. It is also known that serotonergic dorsal raphe cells, which send projections to the prefrontal cortex, striatum, and nucleus accumbens (Di Matteo et al. 2008), are controlled by cortical pyramidal cells (Martin-Ruiz et al. 2001). In addition, descending glutamatergic pathways may indirectly inhibit raphe cells by stimulation of GABAergic interneurons (Di Matteo et al. 2008; Martin-Ruiz et al. 2001). Desensitization of 5-HT_2A_ receptors by repeated administration of 25I-NBOMe may exert a weaker effect on GABA interneurons in comparison to a single dose leading to higher extracellular levels of 5-HT in response to a challenge dose, as it is observed in the striatum and nucleus accumbens. Moreover, it needs to be mentioned that basal 5-HT levels in the striatum of rats treated repeatedly with 25I-NBOMe decreased in comparison to the control group (Table 2S, supplementary material). Therefore, the response of local 5-HT_2A_ receptors to the 25I-NBOMe challenge dose could be stronger to compensate for the reduction in basal neurotransmitter level.

It has been suggested that 5-HT_2A_ receptors localized on cortico-striatal axons can regulate glutamatergic activity in the striatum (Ansah et al. 2011). Similarly, 5-HT_2A_ receptors localized on pyramidal cells projecting to the nucleus accumbens may be responsible for glutamate release in this region (Aghajanian and Marek 1997). Interestingly, in our study, the response of glutamate neurons to a challenge dose was weaker in the striatum but more potent in the nucleus accumbens in rats treated repeatedly with 25I-NBOMe in comparison to the acutely injected group. This divergent effect may be related to inhibitory D_2_ receptors present on cortico-striatal/cortico-accumbal glutamatergic terminals (Cepeda et al. 2001; Del Arco and Mora 2008; Maura et al. 1988). Presynaptic modulation of glutamate release along the cortico-striatal pathway was enhanced in D_2_ receptor knock-out animals; thus, D_2_ receptors function to decrease glutamate release in the dorsal striatum (Cepeda et al. 2001). The decrease in basal DA striatal levels in repeatedly injected rats may affect the sensitivity of D_2_ receptors in their inhibitory control of glutamate release leading to a strong reduction in extracellular glutamate level compared to a single dose (Table 2S, supplementary material). In contrast, an increased response of glutamatergic terminals in the nucleus accumbens to a challenge dose in repeatedly 25I-NBOMe-treated animals compared to a single dose may instead result from disinhibition of glutamate cortico-accumbal neurons from GABAergic control.

### Regulation of ACh release in the frontal cortex, striatum, and nucleus accumbens

Basal forebrain projections constitute a majority of cholinergic innervation to the cortex (Lebois et al. 2018). It generally appears that 5-HT exerts a stimulatory influence on the release of ACh (Saito et al. 1996). On the other hand, 5-HT has been shown to inhibit cholinergic neurons in the pedunculopontine and dorsolateral tegmental neurons, which express 5-HT_2A_ receptors (Koyama and Kayama 1993). Thus, the effect of 5-HT on the cholinergic system depends on the receptor localization, making the regulation very complex. Authors of one study showed that the 5-HT_2A/2C_ agonist DOI as well as mescaline enhanced ACh release in the rat mPFC (Nair and Gudelsky 2004). The striatum and nucleus accumbens contain numerous cholinergic interneurons (Meredith and Wouterlood 1990). It was shown that 5-HT_2A_ receptors mediated depolarization-induced ACh release from cholinergic interneurons in the striatum, but in the ventral part of this region, cholinergic interneurons were hyperpolarized by 5-HT acting at 5-HT_1A/1B_ receptors (Blomeley and Bracci 2005; Bonsi et al. 2007; Virk et al. 2016). Moreover, there are DA and ACh relationships in the striatum and nucleus accumbens, as DA inhibits ACh release acting at D_2_ receptors expressed on cholinergic interneurons, while stimulation of ACh release via D_1_ receptors needs a higher concentration of the agonist (Straub et al. 2014; Wedzony et al. 1988). In our present work, 25I-NBOMe administered repeatedly increased ACh release in the frontal cortex, striatum, and nucleus accumbens in response to a challenge drug dose. In contrast, a single dose of 25I-NBOMe slightly but significantly decreased ACh release in all the brain regions studied. The inhibitory effect of single doses may be mediated indirectly through dopamine D_2_ receptors located in striatal and accumbal cholinergic interneurons and activated by 5-HT_2A_ receptor-mediated DA release. The increase in ACh release by repeated administration of 25I-NBOMe in all the brain regions studied seems to be related to 5-HT_2A_ receptors, as also shown elsewhere (Nair and Gudelsky 2004).

### The effect on animal behavior

25I-NBOMe reduced motor activity of rats evaluated in the OF test. The time of walking and the number of peeping episodes were lower in animals treated repeatedly with 25I-NBOMe than in saline control or acute 25I-NBOMe 0.3 mg/kg groups. Control of animal movement is mediated through both striato-pallidal and striato-nigral GABAergic medium-sized spiny neurons (MSN), which express dopamine D_1_ and D_2_ (Alex and Pehek 2007; Gerfen et al. 1990) and serotonin 5-HT_2A_ receptors (Ansah et al. 2011). However, heteroreceptors located on the terminals of the cortico-striatal glutamatergic axons appear to be the significant source of 5-HT_2A_ receptors (Bubser et al. 2001). Since glutamate and DA inputs terminate on the same MSN spines, these sites offer the potential for physiological interactions between the glutamate and DA transmitter systems (Freund et al. 1984). The state of activation of the D_1_ direct and D_2_ indirect GABAergic pathways depends on the amount of extracellular DA. In turn, DA release depends on 5-HT_2A_-mediated glutamate levels stimulating the striatal NMDA receptors (Cepeda et al. 1993). As evidenced in our study, the disturbed balance between DA and glutamate levels in the striatum by repeated 25I-NBOMe administration may cause insufficient activation of the D_1_ receptor-expressing direct pathway and D_2_ receptor-expressing indirect pathway projecting to the SN pars reticulata and the internal globus pallidus and subthalamic nucleus, respectively (Gerfen et al. 1990) resulting in disrupted rats’ motor performance. Moreover, Del Arco et al. (2007) presented reduced spontaneous motor activity of rats in OF after stimulation of prefrontal D_2_ receptors in relation to DA release in the nucleus accumbens. The decrease in the basal DA levels in the striatum observed in our study may account for the reduction of motor activity of rats in OF after repeated administration of 25I-NBOMe. Significantly, the activity of MSN neurons is also regulated by muscarinic receptors. The direct GABAergic striatal output pathway contains both excitatory M_1_ and inhibitory M_2_ muscarinic receptors, while the indirect pathway primarily has M_1_ receptors (Singer and Minzer 2003). 25I-NBOMe-induced increase in ACh release causing deregulation of striatal output pathways may also contribute to the reduced rats’ motor activity in the OF test. It has to be mentioned that antimuscarinic drugs are administered in the treatment of motor symptoms of Parkinson’s disease (Pisani et al. 2007).

Besides the striatum, where functional glutamate-DA interactions are crucial in motor control, these interactions in the frontal cortex are implicated in working memory and cognition. In our study, the exploration of a novel object in the NOR test was disturbed by all treatments. However, the exploration time of animals pretreated with repeated doses of 25I-NBOMe was longer; yet, animals did not differentiate between the familiar and novel object. The decrease in *Ri* in the group repeatedly treated with 25I-NBOMe suggests a deficit in memory processing. The evidence from several studies has suggested the critical involvement of the 5-HT_2A_ receptors in the prefrontal cortex activity and its cognitive functions, such as working memory and attention (Zhang and Stackman 2015). The role of the 5-HT_2A_ receptor was evidenced by a study in which systemic activation of the 5-HT_2A_ receptor by an agonist significantly enhanced the time the mice spent exploring the new object presented during the test session 24 h later (Zhang et al. 2013). The relevance of the 5-HT_2A_ receptor for object memory processes was also demonstrated by the results of a study proving that the local infusion of the 5-HT_2A_ receptor antagonist MDL 11,939 into the medial prefrontal cortex impaired retrieval of the object-in-context memory in rats (Bekinschtein et al. 2013). These data suggest that 5-HT_2A_ receptor activation potentiates memory consolidation. Furthermore, it was proven that 5-HT_2A_ receptor activation increased the extracellular efflux of glutamate in the dorsal hippocampus suggesting that memory consolidation might result from the potentiation of glutamate release in this region (Zhang and Stackman 2015). The data of Zhang and Stackman Jr. (2015) points out that hippocampus interaction with the frontal cortex also plays an important role in memory performance. Recognition of a novel object seems to depend on a functional interaction between the hippocampus and the perirhinal or medial prefrontal cortices (Barker and Warburton 2011). Therefore, it may be hypothesized that the memory impairment demonstrated in our study in 25I-NBOMe-repeatedly treated rats may result from the decreased activity of 5-HT_2A_ receptor-mediated glutamate release in the frontal cortex in comparison to the acutely injected group. Furthermore, it has been evidenced that ACh cortical function has been linked to the control of circuits underlying attention and memory (Deepak et al. 2012; Picciotto et al. 2012). Havekes et al. (2011) suggest that the striatal cholinergic system is also significant for learning and memory through interactions with dopaminergic and GABAergic systems. It is well established that cholinergic dysfunction accompanies a cognitive decline in Alzheimer’s disease. The increased ACh release observed in our study stands in contradiction to the results of the NOR test showing memory impairment by repeated treatment with 25I-NBOMe. However, the increase in ACh levels is not very potent compared to control and might be less meaningful for memory processes than glutamate decrease.

Halberstadt and Geyer (2018) reported that some phenylethylamine and indoleamine hallucinogens reduced rats’ locomotor activity in unfamiliar environments, which suggested that this effect reflected the fear in novel settings and increased center avoidance induced by hallucinogens reminding agoraphobia observed in humans. Our results from the LDB test agree with the above findings since animals pretreated with single and repeated doses of 25I-NBOMe spent more time in the dark compartment, while the time spent in the light compartment was shorter in comparison to the control rats. These data suggest that 25I-NBOMe administered in either single or repeated doses of 0.3 mg/kg is likely to induce anxiety in animals. Similarly, the reduction of the open arm entries in the elevated plus maze test after subchronic intermittent injections of low doses of ketamine and psilocin points to a mildly anxiogenic behavioral profile (Horsley et al. 2018). However, in contrast to a single dose, 25I-NBOMe administered repeatedly increased vertical and stereotypical activity in the dark zone. These results are contradictory to our findings in the OF test. This difference may result from the setting of the test. The animals seemed to be afraid of the open arena while they were actively exploring closed space, what indicates a biphasic locomotor profile. It may be suggested that the environment may have an impact on the manner in which psychedelics affect animals’ strategies to cope with fear.

The neurochemical data from our study indicate that the changes in neurotransmitter levels induced by 25I-NBOMe may be implicated in symptoms of anxiety in the LDB test. Some research suggests that DA may be related to social phobia and obsessive–compulsive disorder. Low levels of 5-HT are also believed to play a role in anxiety stimulation (Martin et al. 2009). Animal models and human clinical drug trials have demonstrated that drugs, through altering glutamate transmission, produce a potential anxiolytic action in many different paradigms (Cortese and Phan 2005). We observed changes in extracellular DA, 5-HT, and glutamate levels in all studied brain regions under the influence of 25I-NBOMe. The enhanced DA levels in the striatum and nucleus accumbens may be responsible for a marked increase in the vertical and stereotypical activity of animals after repeated treatment with 25I-NBOMe compared to a single dose. On the other hand, the decreased response of 5-HT in the frontal cortex under repeated 25I-NBOMe dosing regimen might be connected with light zone avoidance by rats and may indicate an elevated anxiety level. Desensitization of the 5-HT_2A_ receptor resulting in the decreased response of cortical glutamate pathways and hyperactive limbic neuronal systems may constitute the background mechanism of anxiogenic effects of 25I-NBOMe in the LDB test.

### Conclusions and limitations

In conclusion, repeated treatment with a low dose of 25I-NBOMe induced a decline in WDS and loss of responsiveness of DA, 5-HT, and glutamate neuronal pathways in the rat frontal cortex to a challenge dose of 25I-NBOMe compared to a single administration. The changes in DA, 5-HT, ACh, and glutamate levels in the striatum and nucleus accumbens seem to depend on the activity of the 5-HT_2A_ receptor on cortical pyramidal cells since blockade of this receptor with selective antagonist reversed the effect of 25I-NBOMe, as evidenced in our recent study (Herian et al. 2020). The mechanism of the 25I-NBOMe-induced impairment of several behavioral functions, such as locomotor activity, short-term memory, and anxiety, seems to be more complex and result from a modulatory role of distinct receptor populations in regions controlled by cortical projections. The precise identification of receptors controlling the behavioral response to repeated treatment with a low dose of 25I-NBOMe needs further studies.

## Supplementary Information

Below is the link to the electronic supplementary material.

ESM 1 (DOC 46 kb)

## Declarations

All animal use procedures were conducted in strict accordance with the European regulations for animal experimentation (EU Directive 2010/63/EU on the Protection of Animals Used for Scientific Purposes). The experimental protocols were approved by the Local Ethics Commission for Experimentation on Animals (permit numbers: 186/2017 and 188/2017).
