# Supplementary material for: Tolerance to neurochemical and behavioral effects of the hallucinogen 25I-NBOMe
Source: Psychopharmacology (Berl). 2021 May 25;238(8):2349–64. doi: 10.1007/s00213-021-05860-5 (PMC8292280; doi:10.1007/s00213-021-05860-5)
Supplement: Supplementary file 1 — (DOC 46 kb) [file 213_2021_5860_MOESM1_ESM.doc]

**SUPPLEMENTARY INFORMATION (SI)**

**Tolerance to Neurochemical and Behavioral Effects of the Hallucinogen 25I-NBOMe**

Monika Herian, Mateusz Skawski, Adam Wojtas, Małgorzata K. Sobocińska, Karolina Noworyta, Krystyna Gołembiowska

Maj Institute of Pharmacology, Polish Academy of Sciences, Department of Pharmacology, 31-343 Kraków, 12 Smętna, Poland

Correspondence: Krystyna Gołembiowska, e-mail: nfgolemb@cyf-kr.edu.pl; phone +48 12

662 32 11; fax +48 12 637 45 00

**List of content**

| **Table 1S**………………………………………………………………………………………. | 2 |
| --- | --- |
| **Table 2S**………………………………………………………………………………………. | 2 |

**Table 1S.** Statistical analysis of the area under the curve (AUC) values for dopamine (DA), serotonin (5-HT), glutamate (GLU) and acetylcholine (ACh) in the rat frontal cortex, striatum and nucleus accumbens, presented in the Figure 4.

|  | **Frontal cortex** | **Striatum** | **N. accumbens** |
| --- | --- | --- | --- |
| **DA** | F_2,15_ = 154, p<0.0001 | F_2,15_ = 131, p<0.0001 | F_2,15_ = 746, p<0.0001 |
| **5-HT** | F_2,15_ = 398, p<0.0001 | F_2,15_ = 429, p<0.0001 | F_2,15_ = 412, p<0.0001 |
| **GLU** | F_2,15_ = 83, p<0.0001 | F_2,15_ = 195, p<0.0001 | F_2,15_ = 341, p<0.0001 |
| **ACh** | F_2,15_ = 1433, p<0.0001 | F_2,15_ = 441, p<0.0001 | F_2,15_ = 167, p<0.0001 |

**Table 2S**. Basal levels of dopamine (DA), serotonin (5-HT), acetylcholine (ACh) and glutamate (GLU) in the rat frontal cortex, striatum and nucleus accumbens in saline and 25I-NBOMe (0.3 mg/kg ×7) treated rats.

|  | (nM) | | | (μM) |
| --- | --- | --- | --- | --- |
|  | **DA** | **5-HT** | **ACh** | **GLU** |
| **saline** | | | | |
| Frontal cortex | 1.89 ± 0.16 | 0.46 ± 0.03 | 20.84 ± 2.60 | 2.71 ± 0.21 |
| Striatum | 3.99 ± 0.27 | 0.31 ± 0.04 | 9.60 ± 1.10 | 1.33 ± 0.13 |
| N. accumbens | 1.18 ± 0.06 | 0.36 ± 0.07 | 6.38 ± 1.18 | 1.69 ± 0.12 |
| **25I-NBOMe 0.3 mg/kg ×7** | | | | |
| Frontal cortex | 2.31 ± 0.22 | 0.48 ± 0.05 | 20.77 ± 5.50 | 2.77 ± 0.31 |
| Striatum | 2.64 ± 0.54* | 0.23 ± 0.01* | 8.45 ± 1.70 | 1.36 ± 0.31 |
| N. accumbens | 1.32 ± 0.12 | 0.33 ± 0.04 | 5.16 ± 0.58 | 1.37 ± 0.19 |

For each group n = 6; * *p* < 0.02 saline vs. 25I-NBOMe 0.3 mg/kg ×7 (one-way ANOVA and Tukey’s post hoc test)
